# The causal effect of number of children on later-life overweight and obesity in parous women. An instrumental variable study

**DOI:** 10.1016/j.pmedr.2021.101528

**Published:** 2021-08-18

**Authors:** Thijs van den Broek, Maria Fleischmann

**Affiliations:** a Erasmus School of Health Policy & Management, Erasmus University Rotterdam, Postbus 1738, 3000 DR Rotterdam, The Netherlands; b Department of Health Sciences, Vrije Universiteit Amsterdam, Amsterdam, The Netherlands

**Keywords:** Fertility, Parity, Obesity, Body mass index, Causal inference

## Abstract

Many older women in Europe are overweight or obese. One of the factors linked to overweight and obesity among older women is childbearing. However, results of observational studies on the association between women’s number of children and excess weight should be interpreted with caution, because they may be prone to bias due to residual confounders or reverse causation. We use data of women aged 50 and older with at least two births from seven waves the Survey of Health, Ageing and Retirement in Europe (n = 113,932) collected between 2004 and 2020. We adopt an instrumental variable approach that exploits the well-established preference for mixed-sex offspring to estimate the causal effect of number of children on older parous women’s body mass index (BMI) and their risk of overweight (BMI >= 25 kg/m2) and obesity (BMI >= 30 kg/m2). The instrumental variable models provided evidence for a causal positive effect of having 3 + children as opposed to 2 children on mothers’ body mass index, overweight (BMI >= 25 kg/m2) risk and obesity (BMI >= 30 kg/m2) risk. Predicted BMI was 1.8 kg/m2 higher for mothers with 3 + children than for mothers with 2 children, and their predicted probability of overweight and obesity was 18.3 and 8.6 percentage points higher, respectively. Results remained virtually unchanged after adjusting for age, educational attainment, country and wave of data collection.

## 1 Introduction

In many developed countries, the share of overweight and obese people among the older population is rising (Großschädl and Stronegger, 2019, Molarius et al., 2016, Reinders et al., 2018, Sulander et al., 2004). Peralta et al. (2018) recently noted that “the prevalence of obesity in older European adults has […] reached epidemic proportions” (p. 528). The rise of overweight and obesity in older people has substantial public health implications, because overweight and, particularly, obesity in later life are associated with elevated morbidity risks (Lebenbaum et al., 2018, Ma et al., 2020, Salihu et al., 2009).

One of the factors linked to overweight and obesity among older women is childbearing. Umberson et al. (2011) argue that “parenthood seems to shape a long-term, gradual, and cumulative pattern of weight gain” (p. 1329). Observational studies indeed typically show a positive association between the number of children given birth to and women’s body mass index (Kim et al., 2007a, Kim et al., 2007b, Kravdal et al., 2020, Peters et al., 2016, Umberson et al., 2011, Zoet et al., 2019). This positive association persists in later midlife and old age (Bastian et al., 2005, Bobrow et al., 2013, Peters et al., 2016, Weng et al., 2004).

One potential mechanism underlying the association between number of children and women’s bodyweight is biological. High levels of the hormone progesterone during pregnancy are responsible for bodyfat accumulation during the first and second trimesters of the gestation period (Gunderson and Abrams, 2000). Given that weight gained during pregnancy is often retained postpartum, the accumulation of excess gestational weight gained during successive pregnancies may put mothers with a greater number of children at increased risk of overweight and obesity (Harris et al., 1997). Increased bodyweight at the start of a higher-order pregnancy as a consequence of previous pregnancies is moreover associated with greater gestational weight gain (Harris et al., 1997, Lacroix et al., 2016, Rosenberg et al., 2003).

A second potential underlying mechanism is via lifestyle changes (Gunderson and Abrams, 2000). Older adults with a greater number of children tend to have unhealthier lifestyles (Grundy and Read, 2015, Van den Broek, 2021). As argued by Umberson et al. (2011), “additional children impose more constraints and responsibilities that influence weight change” (p. 1325). Particularly for women having more children may imply more demanding family responsibilities, which, in turn, constitute a barrier for exercise (El Ansari and Lovell, 2009). Consistent with this reasoning, studies have shown that a higher number of children was associated with physical inactivity among women in various contexts (Eyler et al., 2002).

The results of observational studies on the association between women’s number of children and excess weight should be interpreted with caution, because they may be prone to bias due to residual confounders or reverse causation. In developed countries, completed fertility tends, for instance, to be lower, for women who became mothers at a later age and for women with higher educational attainment (Isen and Stevenson, 2010, Kravdal and Rindfuss, 2008). Later transitions to parenthood and higher educational attainment are also associated with lower risks of obesity and overweight (Devaux and Sassi, 2013, Kim, 2016, Peters et al., 2016). Failure to account for any confounding variable of this kind will bias the estimated effect of high fertility on mothers’ excess weight in an observational study. Moreover, overweight and obese women need more time than their non-overweight counterparts to conceive and consequently have lower completed fertility (Pasquali, 2006, Silvestris et al., 2018). This may result in underestimation of the impact of high fertility on overweight and obesity in observational studies.

Drawing data from the Survey of Health, Ageing and Retirement in Europe, we aim to estimate the causal effect of number of children on body mass index, overweight and obesity among older women in Europe. We extend earlier work on the association between fertility and excess weight by adopting a quasi-experimental instrumental variable approach that is less prone to omitted variable bias and to bias due to the impact of overweight and obesity on fertility.

## 2 Data and measures

### 2.1 Sample

Data used were from the Survey of Health, Ageing and Retirement in Europe (SHARE)(Börsch-Supan et al., 2013). SHARE is a longitudinal, cross-national dataset on the health, socioeconomic status and social relations of older people in Europe and Israel.

**Fig. 1:**
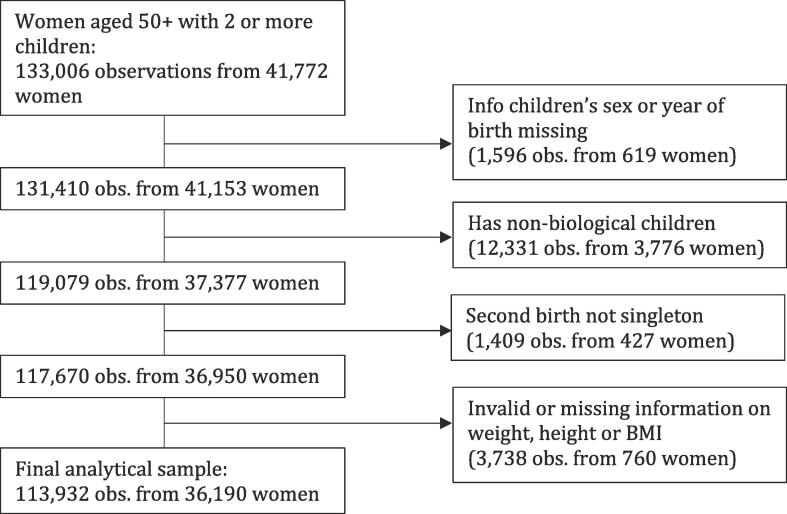
Flow chart for study sample.

Currently, eight waves of data, collected between 2004 and 2020, are available. We used all waves with the exception of Wave 3, because Wave 3 had a considerably different setup than the other waves and because variables relevant for the current study were not collected in this wave. The analytical sample was restricted to 133,006 observations from 41,772 women who were aged 50 or older and had at least 2 children. We then excluded mothers if information about the sex or birth year of at least one of their children was missing. We also dropped observations for mothers who reported having non-biological children and women whose second birth was not singleton, because the instrument used here could not be properly coded for these respondents. Finally, cases with missing or invalid information on weight, height or body mass index were deleted list-wise. This procedure resulted in a final analytical sample of 113,932 observations nested in 36,190 women (see Fig. 1).

### 2.2 Measures

Body Mass Index (BMI) was calculated by dividing respondents’ self-reported weight in kilograms by the square of their self-reported height in meters (kg/m2). Following earlier work (Flegal et al., 2019, Williams, 2003), reported weights lower than 25 kg or greater than 250 kg, reported heights lower than 100 cm or greater than 240 cm and BMI scores lower than 14 kg/m2 or greater than 55 kg/m2 were considered invalid. Following conventional guidelines (World Health Organization, 2020), we coded respondents as overweight when they had a BMI greater than or equal to 25 kg/m2, and as obese when their BMI was greater than or equal to 30 kg/m2.

The central explanatory variable was a dichotomous variable capturing high fertility, here operationalized as having three or more living children. The sex composition of respondents’ two oldest living children was used as an instrument to predict this plausibly endogenous variable. Note that information about deceased children was not available, so the two oldest living children were considered as being respondents’ two firstborn children. The instrument was coded as a dichotomous variable distinguishing between mothers whose two firstborn children had identical sexes (daughter-daughter or son-son) and mothers of whom the two firstborn children had different sexes (daughter-son or son-daughter).

## 3 Methods

**Fig. 2:**
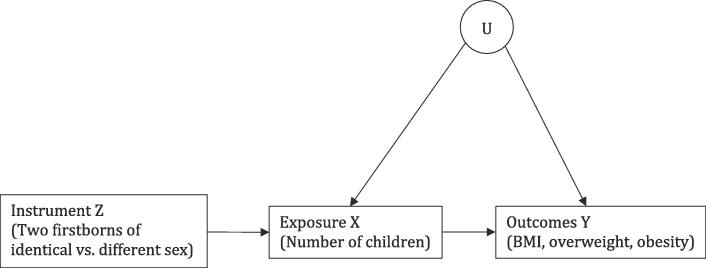
Directed Acyclic Graph.

In order to avoid potential bias due to confounding or reverse causality, we estimated a series of instrumental variable (IV) models (Martens et al., 2006). Instrumental variables can be used to estimate causal effects of an endogenous exposure when the three conditions depicted in Fig. 2 are met (cf. Labrecque and Swanson, 2018). First, the instrument *Z* should be associated with the exposure *X* (the “relevance” condition). Second, the association between the instrument *Z* and the outcome *Y* should not be confounded by omitted variables *U* (the “exchangeability” condition). Finally, the instrument *Z* should affect the outcome *Y* only via the exposure *X* (the “exclusion restriction” condition).

Following prior work on later-life psychological wellbeing (Kruk and Reinhold, 2014, Van den Broek, 2020, Van den Broek and Tosi, 2020), we exploit the well-documented preference for mixed-sex offspring in Europe (Andersson et al., 2006, Hank and Kohler, 2000, Mills and Begall, 2010). This preference makes mothers of two children more inclined to have a third child when the two firstborn children are either both daughters or both sons than when they are a daughter and a son. Nature’s random assignment of whether the sex of the second child is different from the sex of the first child (our instrument) is thus likely to result in exogenous differences in the probability of a third birth (our exposure), in which case both the relevance condition and the exchangeability condition are met. Whether the exclusion restriction condition is met cannot be tested empirically (Labrecque and Swanson, 2018) and hinges on the absence of theoretically plausible pathways from the instrument (sex composition of two firstborn children) to the outcomes (BMI, overweight and obesity) other than via the exposure (number of children).

We estimated the causal bodyweight effects of number of children in a two-stage approach. Equation (1) presents the first stage model:

(1) $${Pr(X}_{i}=1)=\alpha_{0}+\alpha_{1}Z_{i}+\varepsilon_{i}$$

In the first stage, the exogenous instrument *Z*, i.e. whether or not the two firstborn children of mother *i* are of the same sex, was used to predict the probability that mother *i* had a third birth *X*. Coefficient $\alpha_{1}$ denotes the difference in the linear probability estimate of having had a third birth between mothers whose two firstborn children are of the same sex and mothers whose two firstborn children are of different sexes. As shown in Equation (2), this exogenous parity progression difference was subsequently used to estimate the causal effect of having three or more children, as opposed to two, on BMI.

(2) $$Y_{i}=\beta_{0}+\beta_{1}{\hat{X}}_{i}+u_{i}$$

Here, BMI *Y* for mother *i* was regressed on estimated probability of having a third child as predicted in the first stage *X̂*. The model shown in Equation (2) was estimated using two-stage least squares regression. For the models predicting overweight and obesity, a probit regression was performed in the second stage. As shown in Equation (3), the probability of overweight and obesity, respectively, *Y* for mother *i* was regressed on *X̂*, i.e. the probability of having a third child as predicted in the first stage.

(3) $$Pr\left(Y_{i}=1\right)={\Phi (\beta }_{0}+\beta_{1}{\hat{X}}_{i}+u_{i})$$

All models were estimated with robust standard errors to account for the nested nature of the data.

## 4 Results

### 4.1 Descriptive statistics

Table 1 shows characteristics of the sample. Approximately four out of ten mothers in the sample had a third birth. The average BMI was 26.8 kg/m2. Note that this average value is well above the conventional threshold for overweight. Approximately six out of ten respondents were overweight, as indicated by a BMI of 25 or higher, and almost one in four was obese, i.e. had a BMI of 30 or higher.

**Table 1:** Sample characteristics; means and percentages.

	All		2 children		3 + children	
	% (n) / M	(SD)	% (n) / M	(SD)	% (n) / M	(SD)
Third birth	41.0% (46,722)					
Body Mass Index	26.8	(4.9)	26.7	(4.8)	27.1	(5.1)
Overweight (BMI>=25 kg/m2)	60.2% (68,632)		59.0% (39,656)		62.0% (28,976)	
Obese(BMI>=30 kg/m2)	23.1% (26,295)		21.5% (14,465)		25.3% (11,830)	
Age	67.5	(10.1)	66.9	(9.9)	68.5	(10.3)
Country:						
Austria	5.2% (5,952)		4.9% (3,258)		5.8% (2,694)	
Germany	5.4% (6,126)		5.6% (3,739)		5.1% (2,387)	
Sweden	5.3% (6,041)		5.2% (3,463)		5.5% (2,578)	
Netherlands	6.1% (6,995)		5.8% (3,928)		6.6% (3,067)	
Spain	7.6% (8,630)		6.4% (4,327)		9.2% (4,303)	
Italy	6.9% (7,838)		7.2% (4,860)		6.4% (2,978)	
France	7.3% (8,267)		6.1% (4,127)		8.9% (4,140)	
Denmark	4.9% (5,549)		5.0% (3,371)		4.7% (2,178)	
Greece	4.3% (4,909)		5.3% (3,571)		2.9% (1,338)	
Switzerland	4.2% (4,733)		4.0% (2,678)		4.4% (2,055)	
Belgium	7.3% (8,309)		6.6% (4,433)		8.3% (3,876)	
Israel	3.7% (4,228)		1.9% (1,246)		6.4% (2,982)	
Czech Republic	7.9% (9,037)		9.5% (6,396)		5.7% (2,641)	
Poland	3.4% (3,858)		2.8% (1,875)		4.2% (1,983)	
Ireland	0.2% (2 8 2)		0.1% (66)		0.5% (2 1 6)	
Luxembourg	1.2% (1,377)		1.3% (9 0 0)		1.0% (4 7 7)	
Hungary	1.2% (1,378)		1.5% (1,034)		0.7% (3 4 4)	
Portugal	1.1% (1,258)		1.2% (7 8 4)		1.0% (4 7 4)	
Slovenia	4.8% (5,418)		5.8% (3,904)		3.2% (1,514)	
Estonia	7.0% (7,985)		8.0% (5,398)		5.5% (2,587)	
Croatia	1.4% (1,634)		1.8% (1,179)		1.0% (4 5 5)	
Lithuania	0.8% (9 3 0)		1.0% (6 5 3)		0.6% (2 7 7)	
Bulgaria	0.5% (5 5 6)		0.7% (4 7 4)		0.2% (82)	
Cyprus	0.3% (3 0 4)		0.2% (1 3 2)		0.4% (1 7 2)	
Finland	0.4% (4 6 4)		0.4% (2 7 1)		0.4% (1 9 3)	
Latvia	0.4% (4 0 0)		0.5% (3 1 8)		0.2% (82)	
Malta	0.3% (3 5 6)		0.2% (1 6 8)		0.4% (1 8 8)	
Romania	0.6% (6 3 2)		0.5% (3 5 1)		0.6% (2 8 1)	
Slovakia	0.4% (4 8 6)		0.5% (3 0 6)		0.4% (1 8 0)	
Wave:						
Wave 1	7.5% (8,555)		6.6% (4,430)		8.8% (4,125)	
Wave 2	9.8% (11,165)		8.9% (5,998)		11.1% (5,167)	
Wave 4	15.2% (17,266)		15.2% (10,220)		15.1% (7,046)	
Wave 5	17.2% (19,613)		17.0% (11,451)		17.5% (8,162)	
Wave 6	19.0% (21,666)		19.6% (13,140)		18.2% (8,526)	
Wave 7	18.6% (21,192)		19.3% (12,970)		17.6% (8,222)	
Wave 8	12.7% (14,475)		13.4% (9,001)		11.7% (5,474)	
Observations	113,932		67,210		46,772	
Respondents	36,190		21,539		14,651	

Note: Data are from the Survey of Health, Ageing and Retirement in Europe (Waves 1, 2, 4, 5, 6, 7, 8).

Compared to their counterparts with two children, women with three or more children had a significantly higher BMI (*F*(1, 113930) = 249.0, *p* < .001). They were also more often overweight (62.0% vs 59.0%; χ^2^(1, *N* = 113932) = 104.5, *p* < .001) and obese (25.3% vs 21.5%; χ^2^(1, *N* = 113932) = 223.5, *p* < .001). It should be noted that these differences may reflect compositional differences between mothers with two children and mothers with three or more children. The latter group was for instance significantly older (*F*(1, 113930) = 722.6, *p* < .001).

### 4.2 Instrumental variable models

The results of the IV models are presented in Table 2. The first stage model showed that the probability of a third birth was almost six percentage points lower for mothers whose two firstborn children were of different sexes than for their counterparts whose two firstborn children were of the same sex. This difference in parity progression is consistent with the preference for mixed sex offspring noted in many European countries (Andersson et al., 2006, Hank and Kohler, 2000, Mills and Begall, 2010). The F-statistic greatly exceeded 10 (*F*(1, 113930) = 418.0, *p* < .001), indicating that sex composition of the two firstborn children is sufficiently predictive of the likelihood of a third birth. This result confirms that the relevance condition for IV models is met.

**Table 2:** Results of two stage least squares and IV probit regression models of Body Mass Index (BMI), overweight and obesity.

	First stage		Second stage					
	Third birth		Body Mass Index (BMI)		Overweight (BMI>=25 kg/m2)		Obesity (BMI>=30 kg/m2)	
	Coeff.	(SE)	Coeff.	(SE)	Coeff.	(SE)	Coeff.	(SE)
								
Third birth			1.848***	(0.492)	0.485***	(0.119)	0.284*	(0.136)
								
Sex composition two firstborn children:								
Identical	Ref.							
Different	−0.059***	(0.003)						
								
Constant	0.440***	(0.002)	26.087***	(0.203)	0.055	(0.052)	−0.852***	(0.052)
								

Notes: Data are from the Survey of Health, Ageing and Retirement in Europe (Waves 1, 2, 4, 5, 6, 7, 8); n = 113,932; Robust standard errors;

* *p* < .05, ** *p* < .01, *** *p* < .001.

The second-stage results showed that high fertility had a causal positive effect on mothers’ BMI in later life. The predicted BMI was 1.8 kg/m2 (*p* < .001) higher for mothers of 3 + children than for their counterparts with 2 children. The IV-probit models indicated that high fertility also was a causal risk factor for overweight and for obesity. Compared to mothers of two children, mothers of 3 + children were 0.49 Z-scores higher (*p* < .001) on the cumulative standard normal distribution of the probability of overweight and 0.28 Z-scores higher (*p* < .05) on the cumulative standard normal distribution of the probability of obesity.

**Fig. 3:**
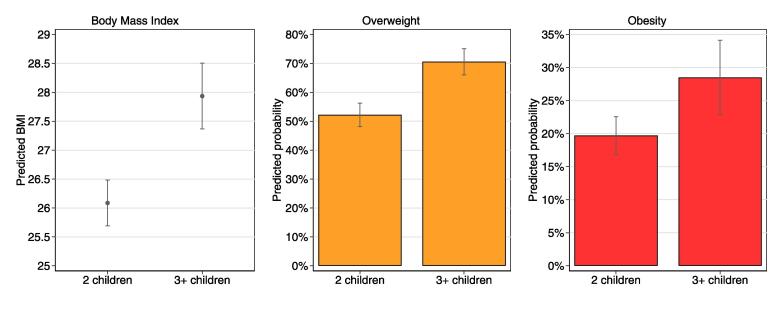
Predicted BMI, overweight risk and obesity risk by completed fertility.

To facilitate an easier interpretation of the IV-results, predicted BMI-scores and predicted probabilities of overweight and obesity for mothers of 2 and mothers of 3 + children are presented in Fig. 3. These adjusted predictions were calculated using the margins command in Stata 16.1 (Williams, 2012). The predicted probability of overweight was 18.3 percentage points higher for mothers with 3 + children than for their counterparts with 2 children (95% CI: 0.099, 0.267; *p* < .001). The predicted probability difference in obesity between both groups of mothers was 8.6 percentage points (95% CI: 0.005, 0.167; *p* < .05).

**Fig. 4:**
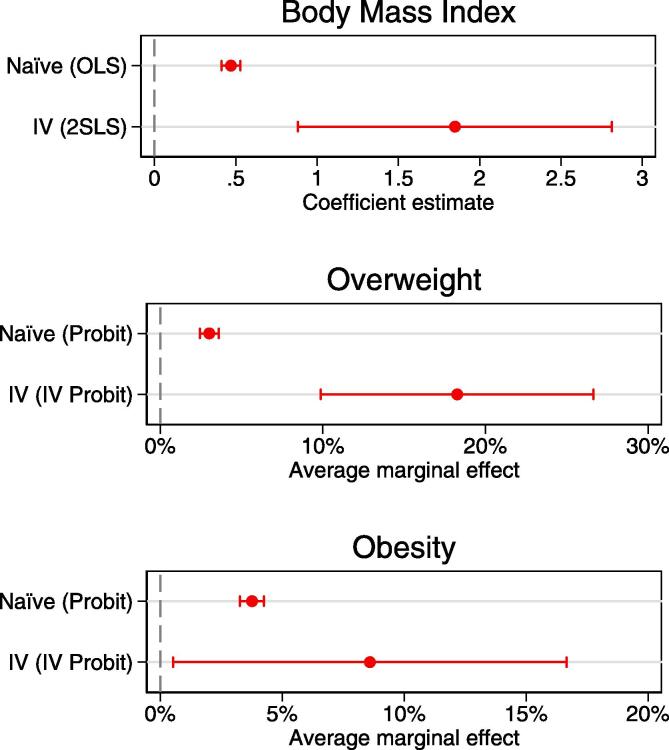
Comparison of naïve and IV estimates of the effects of having 3 + vs 2 children.

Fig. 4 provides an overview of how estimates from naïve models – that is, models in which the outcome of interest is regressed on observed information on whether or not the respondent had a third birth – compares to estimates from the IV models reported here. Estimates of the former models are more efficient, meaning that confidence intervals are narrower, but they may be inconsistent due to omitted variable bias or reversed causality. The latter models produce unbiased estimates of the causal effect of number of children, but these estimates are less precise as indicated by the large confidence intervals. As is clearly visible, the IV estimate of the causal effect of number of children on BMI is considerably larger in magnitude than the estimate of the naïve linear regression model. A robust score chi square test indicated that the exogeneity assumption of the naïve model is violated (χ^2^(1, *N* = 113932) = 8.0, *p* < .001) and thus that the estimates of this model are biased. Similarly, the figure shows that the marginal effect of having 3 + versus 2 children on the probability of obesity is considerably larger in the IV model than in the naïve probit model, and, again, the confidence intervals of the two estimates do not overlap. This indicates that the estimates from the naïve probit model are also biased, which is confirmed by the Wald test of exogeneity (χ^2^(1, *N* = 113932) = 10.9, *p* < .001). With regard to the risk of obesity, the magnitude of the marginal effect of having 3 + versus 2 children is larger in the IV model than in the naïve probit model, but here the confidence intervals overlap. The Wald test of exogeneity was also not statistically significant (χ^2^(1, *N* = 113932) = 1.4, *p* = .243), indicating that we do not have evidence that the naïve probit model is biased.

### 4.3 Robustness checks

Several checks were performed to assess the robustness of our results. Results of these additional analyses are presented in the online supplementary material. Firstly, we checked for violations of the exchangeability condition. We assessed whether mothers whose two firstborn children were of different sexes and their counterparts whose two firstborn children were of the same sex differed systematically with regard to two important predictors of overweight and obesity (educational attainment and age) as well as with regard to their distribution across the countries and waves in our analytical sample. The results are presented in Appendix A. As was to be expected due to the random assignment by nature of our instrument, no systematic differences were found with regard to the age distribution and distribution across waves. Remarkably, however, small, but statistically significant, differences between the two groups distinguished by the instrument used were found with regard to the distribution across educational attainment categories and country. We therefore estimated models adjusted for age, age squared, educational attainment, country and wave (cf. Peytremann-Bridevaux et al., 2007). As shown in Appendix B, the addition of these variables to the models did not change the IV-estimates of the causal effect of high fertility substantially.

Secondly, we estimated models using an alternative instrument specification that distinguished between mothers with two sons and mothers with two daughters as their firstborn children (see Appendix C). Results were again largely similar, but the estimated causal effect of high fertility on obesity was no longer statistically significant when using this less parsimonious specification.

## 5 Discussion

The current study extended earlier work on the links between women’s fertility and later-life overweight and obesity by adopting an instrumental variable approach that produces estimates that are not biased by omitted confounding variables or reversed causation. The analyses presented here provided evidence that high fertility, operationalized as having 3 + children as opposed to 2, had a causal positive effect on women’s BMI in later life. The results also indicated that high fertility was a causal risk factor for overweight and obesity in older women. The current study extends earlier work on the association between the number of children given birth to and women’s bodyweight in later midlife and old age (e.g., Bastian et al., 2005, Bobrow et al., 2013, Peters et al., 2016, Weng et al., 2004) by providing more convincing evidence that this association is causal.

The current study had some notable limitations. Firstly, it relied on self-reports of weight and height to calculate the BMI. Stommel and Schoenborn (2009) compared the concordance between BMI-scores based on self-reported weight and height and BMI-scores based on measured height and weight using 2001–2006 data from the National Health and Nutrition Examination Survey (NHANES) and the National Health Interview Survey (NHIS). They found that the proportion of self-reported 'overweight' or 'obese' persons who actually were overweight or obese based on measured height and weight were 95.8% and 93.9%, respectively. Drawing on data from a Swiss population-based sample, Dauphinot et al. (2009) concluded that misclassification of obesity when using self-reports of weight and height to calculate the BMI was substantially less likely when an alternative obesity threshold of BMI>=29.2 kg/m2 was used. They subsequently validated this alternative threshold in a French population sample. We re-estimated the model of obesity whereby we followed recommendations by Dauphinot et al. (2009) and considered respondents with a BMI of 29.2 or higher to be obese (See Appendix D). Also when using this alternative obesity threshold, the IV-probit model showed that having 3 or more children as opposed to 2 was associated with a causal later-life obesity risk increase. The predicted probability of obesity as indicated by a BMI of 29.2 or higher was 12.1 percentage points (95% CI: 0.038, 0.204; *p* < .01) higher for mothers with 3 + children than for their counterparts with 2 children.

The IV approach adopted here enabled unbiased estimation of the effect of high fertility on overweight and obesity, but this benefit came at the expense of precision. Despite the large analytical sample, the confidence intervals of the estimates presented here were relatively wide. We could therefore only present average treatment effects. This is unfortunate, because heterogeneous treatment effects may be expected, as other research suggested that the effects of high fertility on excess weight may differ between countries and vary by household wealth and ethnicity (Gunderson and Abrams, 2000, Kim et al., 2007b, Kim et al., 2007a). Future studies drawing on even larger samples may explore such variation in the causal effect of high fertility on overweight and obesity in later life.

Finally, information about deceased children was not collected in the SHARE waves used in our analyses. Both our exposure variable (having 3 + versus 2 children) and the instrument used (sex composition of two firstborn children) were therefore based on reports on living biological children. This has likely resulted in measurement error due to misclassification of people with deceased children. However, we have no theoretical reasons to expect these misclassifications to be systematically associated with our instrumental variable. Therefore, this data limitation is in our view unlikely to have biased our results substantially.

The finding that, overall, high fertility has a positive causal effect on mothers’ risk of overweight and obesity in later life is worth considering in the light of the epidemic of later-life overweight and obesity (Peralta et al., 2018). As described earlier, both biological and lifestyle related mechanisms may underlie the causal impact of women’s number of children on bodyweight, overweight and obesity. Interestingly, observational studies by Weng et al., 2004, Kravdal et al., 2020 showed that number of children was not just associated with overweight and obesity in women, but also in men, which could be indicative of a non-biological pathway. We therefore repeated our analyses among male SHARE respondents (see Appendix E). Although no statistically significant evidence for a causal effect of number of children on BMI or the risk of obesity was found among fathers, the analyses indicated that the risk of later-life overweight was 19.4 percentage higher for fathers of 3 + children than for fathers of 2 children points (95% CI: 0.091, 0.297; *p* < .001). The finding that number of children has a causal effect on the risk of overweight not just among mothers, but also among fathers suggests that the mechanism linking fertility history to later-life overweight risk is not exclusively biological and that lifestyle changes also play a role. Consistent with this reasoning, Grundy and Read (2015) reported that high fertility was associated with physical inactivity not just for women but also for men. This suggests that it may be worthwhile for public health practitioners to develop healthy lifestyle interventions targeted specifically to parents with larger families.

## 6 Ethics statement

During Waves 1 to 4, SHARE was reviewed and approved by the Ethics Committee of the University of Mannheim. Wave 4 of SHARE and the continuation of the project were reviewed and approved by the Ethics Council of the Max Planck Society.

## CRedit authorship contribution statement

TvdB was responsible for the conceptualization of the study. MF and TvdB are jointly responsible for the methodology, formal analysis, visualization, the writing of the original draft and the editing of the revised version of the manuscript.

## Declaration of Competing Interest

The authors declare that they have no known competing financial interests or personal relationships that could have appeared to influence the work reported in this paper.
