# Supplementary material for: The causal effect of number of children on later-life overweight and obesity in parous women. An instrumental variable study
Source: Prev Med Rep. 2021 Aug 18;24:101528. doi: 10.1016/j.pmedr.2021.101528 (PMC8683859; doi:10.1016/j.pmedr.2021.101528)
Supplement: Supplementary Data 1 [file mmc1.pdf]

Appendix A. Sample characteristics by instrument category; means and percentages.

	Two firstborns identical sex		Two firstborns different sex		Test of difference
	% (n) / M	(SD)	% / M	(SD)	
Third birth	44.0% (25,118)		38.0% (n=21,604)		$\chi^2(1, N=113{,}932)=$ 415.8, $p < .001$
Body Mass Index	26.9	(4.9)	26.8	(4.9)	$F(1, 113930)=$ 14.3, $p < .001$
Overweight (BMI>=25 kg/m2)	60.8% (34,732)		59.7% (33,900)		$\chi^2(1, N=113{,}932)=$ 15.3, $p < .001$
Obese (BMI>=30 kg/m2)	23.3% (13,331)		22.8% (12,964)		$\chi^2(1, N=113{,}932)=$ 4.3, $p < .05$
Age	67.5	(10.0)	67.6	(10.1)	$F(1, 113{,}930)=$ 1.9, $p = .166$
Education:					$\chi^2(3, N=113{,}932)=$ 25.2, $p < .001$
Low (ISCED 0-2)	45.6% (26,075)		47.1% (26,771)		
Mid (ISCED 3-4)	34.9% (19,950)		34.1% (19,368)		
High (ISCED 5-6)	18.7% (10,653)		18.0% (10,254)		
Missing	0.8% (442)		0.7% (419)		
Country:					$\chi^2(28, N=113{,}932)=$ 78.3, $p < .001$
Austria	5.2% (2,990)		5.2% (2,962)		
Germany	5.3% (3,013)		5.5% (3,113)		
Sweden	5.2% (2,958)		5.4% (3,083)		
Netherlands	6.0% (3,442)		6.3% (3,553)		
Spain	7.7% (4,381)		7.5% (4,249)		
Italy	6.7% (3,854)		7.0% (3,984)		
France	7.5% (4,268)		7.0% (3,999)		
Denmark	4.8% (2,722)		5.0% (2,827)		
Greece	4.2% (2,375)		4.5% (2,534)		
Switzerland	4.2% (2,406)		4.1% (2,327)		
Belgium	7.3% (4,171)		7.3% (4,138)		
Israel	3.6% (2,065)		3.8% (2,163)		
Czech Republic	7.8% (4,481)		8.0% (4,556)		
Poland	3.5% (1,973)		3.3% (1,885)		
Ireland	0.3% (146)		0.2% (136)		
Luxembourg	1.3% (730)		1.1% (647)		
Hungary	1.2% (709)		1.2% (669)		
Portugal	1.0% (598)		1.2% (660)		
Slovenia	4.8% (2,719)		4.8% (2,699)		
Estonia	7.3% (4,197)		6.7% (3,788)		
Croatia	1.5% (832)		1.4% (802)		
Lithuania	0.8% (485)		0.8% (445)		
Bulgaria	0.4% (243)		0.6% (313)		
Cyprus	0.3% (154)		0.3% (150)		
Finland	0.4% (249)		0.4% (215)		
Latvia	0.3% (198)		0.4% (202)		
Malta	0.3% (181)		0.3% (175)		
Romania	0.6% (335)		0.5% (297)		
Slovakia	0.4% (245)		0.4% (241)		

Table continued on next page

Table continued from previous page

Wave:			$\chi^2(6, N=113,932)=$ 4.3, p = .640
Wave 1	7.4% (4,239)	7.6% (4,316)	
Wave 2	9.7% (5,527)	9.9% (5,638)	
Wave 4	15.2% (8,709)	15.1% (8,557)	
Wave 5	17.2% (9,858)	17.2% (9,755)	
Wave 6	19.0% (10,858)	19.0% (10,808)	
Wave 7	18.7% (10,678)	18.5% (10,514)	
Wave 8	12.7% (7,251)	12.7% (7,224)	
Observations	57,120	56,812	
Respondents	18,131	18,059	

Note: Data are from the Survey of Health, Ageing and Retirement in Europe (Waves 1, 2, 4, 5, 6, 7, 8).

Appendix B-1. Results of two stage least squares and IV probit regression models of Body Mass Index (BMI), overweight and obesity.

	First stage		Second stage					
	Third birth		Body Mass Index (BMI)		Overweight (BMI>=25 kg/m2)		Obesity (BMI>=30 kg/m2)	
	Coeff.	(SE)	Coeff.	(SE)	Coeff.	(SE)	Coeff.	(SE)
Third birth			1.800***	(0.464)	0.508***	(0.119)	0.287*	(0.136)
Sex composition two firstborn children:								
Identical	Ref.							
Different	-0.061***	(0.003)						
Age [a]	0.003***	(0.001)	0.122***	(0.005)	0.037***	(0.002)	0.022***	(0.002)
Age [a] squared	-0.000	(0.000)	-0.004***	(0.000)	-0.001***	(0.000)	-0.001***	(0.000)
Education:								
Low (ISCED 0-2)	Ref.		Ref.		Ref.		Ref.	
Mid (ISCED 3-4)	-0.117***	(0.003)	-0.803***	(0.065)	-0.148***	(0.019)	-0.196***	(0.020)
High (ISCED 5-6)	-0.105***	(0.004)	-1.824***	(0.064)	-0.399***	(0.021)	-0.430***	(0.021)
Missing	0.003	(0.017)	-0.223	(0.174)	-0.101*	(0.043)	0.053	(0.046)
Country:								
Austria	Ref.		Ref.		Ref.		Ref.	
Germany	-0.035***	(0.009)	0.266**	(0.090)	0.073**	(0.023)	0.034	(0.026)
Sweden	-0.027**	(0.009)	-0.586***	(0.084)	-0.090***	(0.024)	-0.191***	(0.028)
Netherlands	-0.029**	(0.009)	-0.611***	(0.085)	-0.121***	(0.023)	-0.187***	(0.026)
Spain	-0.009	(0.008)	0.320***	(0.082)	0.157***	(0.022)	0.019	(0.024)
Italy	-0.111***	(0.008)	-0.657***	(0.097)	-0.080**	(0.026)	-0.183***	(0.030)
France	0.035***	(0.008)	-0.567***	(0.085)	-0.158***	(0.022)	-0.095***	(0.025)
Denmark	-0.042***	(0.009)	-0.747***	(0.089)	-0.156***	(0.025)	-0.138***	(0.029)
Greece	-0.206***	(0.009)	0.975***	(0.133)	0.343***	(0.033)	0.101**	(0.039)
Switzerland	-0.006	(0.010)	-1.353***	(0.094)	-0.315***	(0.025)	-0.268***	(0.029)
Belgium	0.010	(0.008)	-0.363***	(0.082)	-0.079***	(0.022)	-0.056*	(0.025)
Israel	0.255***	(0.010)	0.067	(0.154)	0.016	(0.041)	0.004	(0.045)
Czech Republic	-0.163***	(0.008)	1.564***	(0.111)	0.424***	(0.027)	0.301***	(0.031)
Poland	0.065***	(0.010)	1.012***	(0.107)	0.251***	(0.029)	0.237***	(0.030)
Ireland	0.311***	(0.026)	-0.477	(0.346)	-0.165	(0.086)	-0.046	(0.099)
Luxembourg	-0.105***	(0.015)	-0.036	(0.153)	0.035	(0.040)	0.002	(0.044)
Hungary	-0.201***	(0.013)	1.489***	(0.174)	0.435***	(0.045)	0.321***	(0.048)
Portugal	-0.115***	(0.015)	0.507**	(0.163)	0.164***	(0.043)	0.062	(0.045)
Slovenia	-0.169***	(0.009)	0.930***	(0.118)	0.315***	(0.030)	0.117***	(0.035)
Estonia	-0.111***	(0.008)	2.129***	(0.100)	0.469***	(0.024)	0.438***	(0.027)
Croatia	-0.166***	(0.013)	1.125***	(0.152)	0.397***	(0.041)	0.200***	(0.044)
Lithuania	-0.123***	(0.016)	3.077***	(0.193)	0.720***	(0.050)	0.588***	(0.049)
Bulgaria	-0.290***	(0.016)	0.892***	(0.248)	0.292***	(0.067)	0.097	(0.074)
Cyprus	0.099***	(0.028)	0.495	(0.304)	0.114	(0.078)	0.030	(0.081)
Finland	-0.014	(0.023)	1.061***	(0.239)	0.275***	(0.062)	0.274***	(0.066)
Latvia	-0.206***	(0.022)	3.436***	(0.297)	0.746***	(0.075)	0.629***	(0.072)
Malta	0.079**	(0.027)	1.761***	(0.318)	0.321***	(0.077)	0.330***	(0.073)
Romania	-0.012	(0.020)	1.526***	(0.226)	0.415***	(0.058)	0.320***	(0.056)
Slovakia	-0.025	(0.023)	0.866***	(0.232)	0.257***	(0.061)	0.221***	(0.064)

Table continued on next page

Table continued from previous page								
Wave:								
Wave 1	Ref.		Ref.		Ref.		Ref.	
Wave 2	-0.015*	(0.007)	0.083	(0.068)	0.019	(0.019)	0.013	(0.021)
Wave 4	-0.021**	(0.007)	0.194**	(0.065)	0.042*	(0.018)	0.050*	(0.020)
Wave 5	-0.028***	(0.006)	0.197**	(0.063)	0.025	(0.017)	0.059**	(0.019)
Wave 6	-0.036***	(0.006)	0.262***	(0.064)	0.037*	(0.017)	0.084***	(0.019)
Wave 7	-0.042***	(0.006)	0.262***	(0.065)	0.030	(0.018)	0.084***	(0.020)
Wave 8	-0.047***	(0.007)	0.314***	(0.071)	0.032	(0.019)	0.107***	(0.021)
Constant	0.529***	(0.010)	25.814***	(0.250)	-0.140*	(0.065)	-0.852***	(0.052)

Notes: Data are from the Survey of Health, Ageing and Retirement in Europe (Waves 1, 2, 4, 5, 6, 7, 8); n=113,932; Robust standard errors; [a] Centered on age 50;
* $p < .05$, ** $p < .01$, *** $p < .001$

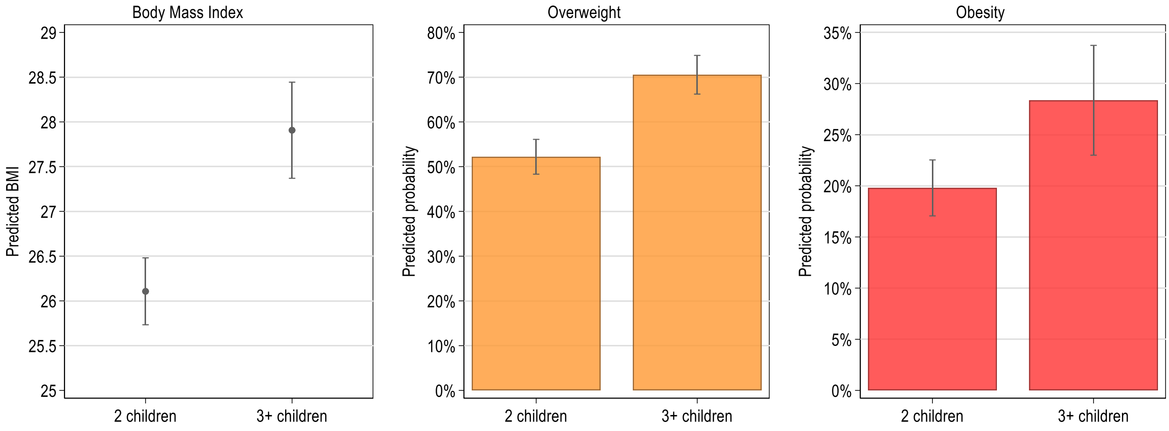

Appendix B-2. Predicted BMI, overweight risk and obesity risk by completed fertility; based on adjusted models.

Appendix C-1. Results of two stage least squares and IV probit regression models of Body Mass Index (BMI), overweight and obesity.

	First stage		Second stage					
	Third birth		Body Mass Index (BMI)		Overweight (BMI>=25 kg/m2)		Obesity (BMI>=30 kg/m2)	
	Coeff.	(SE)	Coeff.	(SE)	Coeff.	(SE)	Coeff.	(SE)
Third birth			1.683***	(0.478)	0.435***	(0.119)	0.220	(0.135)
Sex composition two firstborn children:								
Daughter and son	Ref.							
Two sons	0.050***	(0.004)						
Two daughters	0.070***	(0.004)						
Constant	0.380***	(0.002)	26.155***	(0.197)	0.077	(0.052)	-0.827***	(0.052)

Notes: Data are from the Survey of Health, Ageing and Retirement in Europe (Waves 1, 2, 4, 5, 6, 7, 8); n=113,932; Robust standard errors;
* $p < .05$, ** $p < .01$, *** $p < .001$

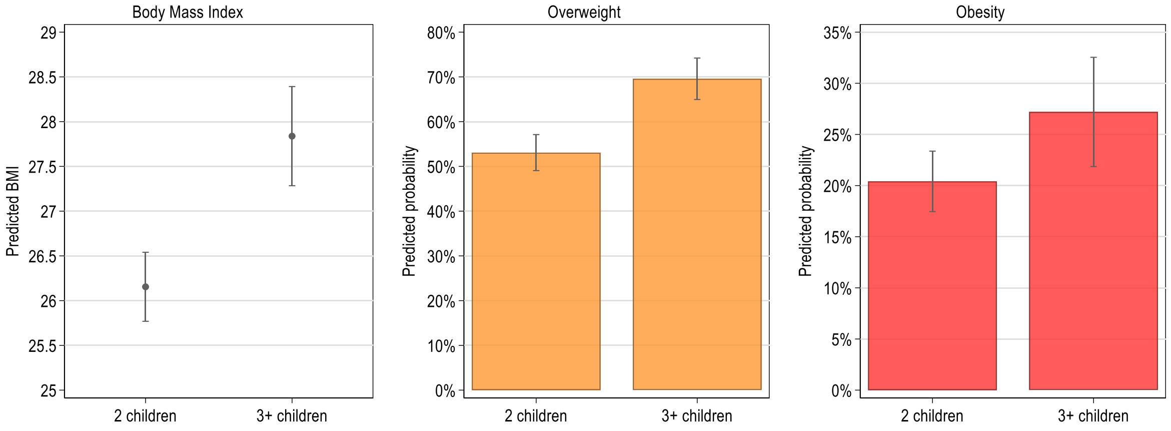

Appendix C-2. Appendix B-2. Predicted BMI, overweight risk and obesity risk by completed fertility; based on model with alternative IV specification.

Appendix D-1. Results of IV probit regression model overweight weight (alternative threshold: BMI >=29.2 kg/m2).

	First stage		Second stage	
	Third birth		Overweight (BMI>=29.2 kg/m2)	
	Coeff.	(SE)	Coeff.	(SE)
Third birth			0.364**	(0.130)
Sex composition two firstborn children:				
Identical	Ref.			
Different	-0.059***	(0.003)		
Constant	0.440***	(0.002)	-0.734***	(0.049)

Notes: Data are from the Survey of Health, Ageing and Retirement in Europe (Waves 1, 2, 4, 5, 6, 7, 8); n=113,932; Robust standard errors;
* $p < .05$, ** $p < .01$, *** $p < .001$

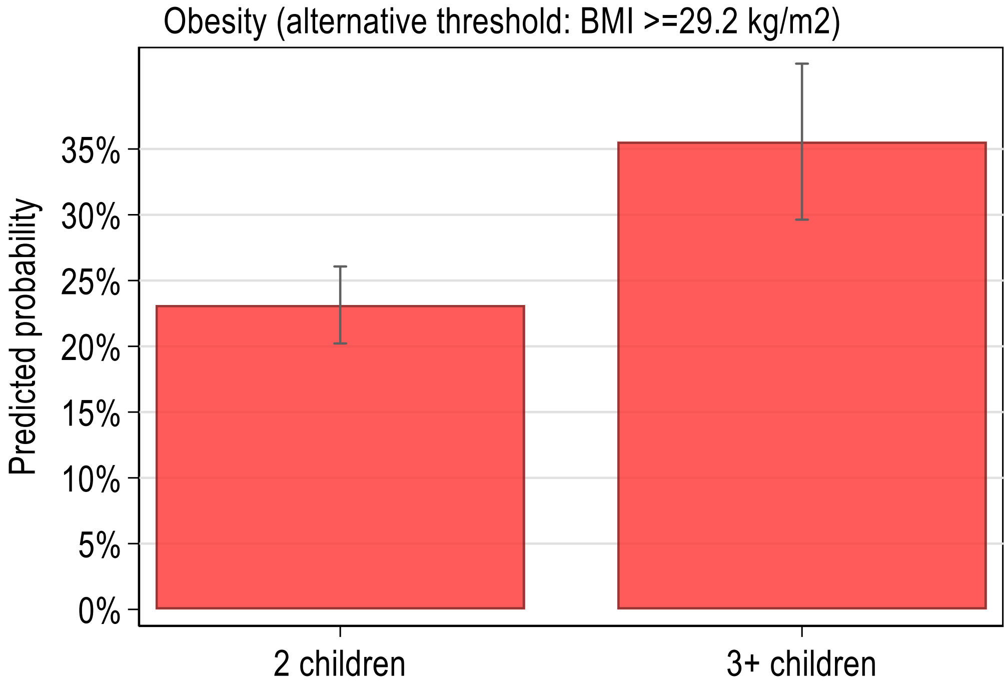

Appendix D-2. Predicted overweight risk (alternative threshold: BMI >=29.2 kg/m2) by completed fertility.

Appendix E-1. Results of two stage least squares and IV probit regression models of Body Mass Index (BMI), overweight and obesity among fathers.

	First stage		Second stage					
	Third birth		Body Mass Index (BMI)		Overweight (BMI>=25 kg/m2)		Obesity (BMI>=30 kg/m2)	
	Coeff.	(SE)	Coeff.	(SE)	Coeff.	(SE)	Coeff.	(SE)
Third birth			0.805	(0.522)	0.555***	(0.156)	0.199	(0.186)
Sex composition two firstborn children:								
Identical	Ref.							
Different	-0.053***	(0.003)						
Constant	0.428***	(0.002)	26.759***	(0.210)	0.237**	(0.073)	-0.920***	(0.069)

Notes: Data are from the Survey of Health, Ageing and Retirement in Europe (Waves 1, 2, 4, 5, 6, 7, 8); n=82,421; Robust standard errors;
* $p < .05$, ** $p < .01$, *** $p < .001$

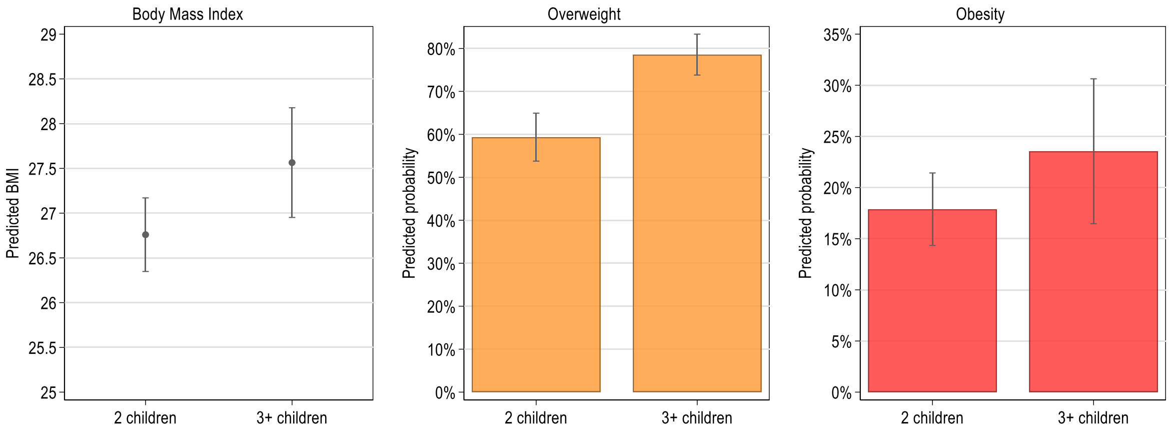

Appendix E-2. Predicted BMI, overweight risk and obesity risk by completed fertility among fathers.
